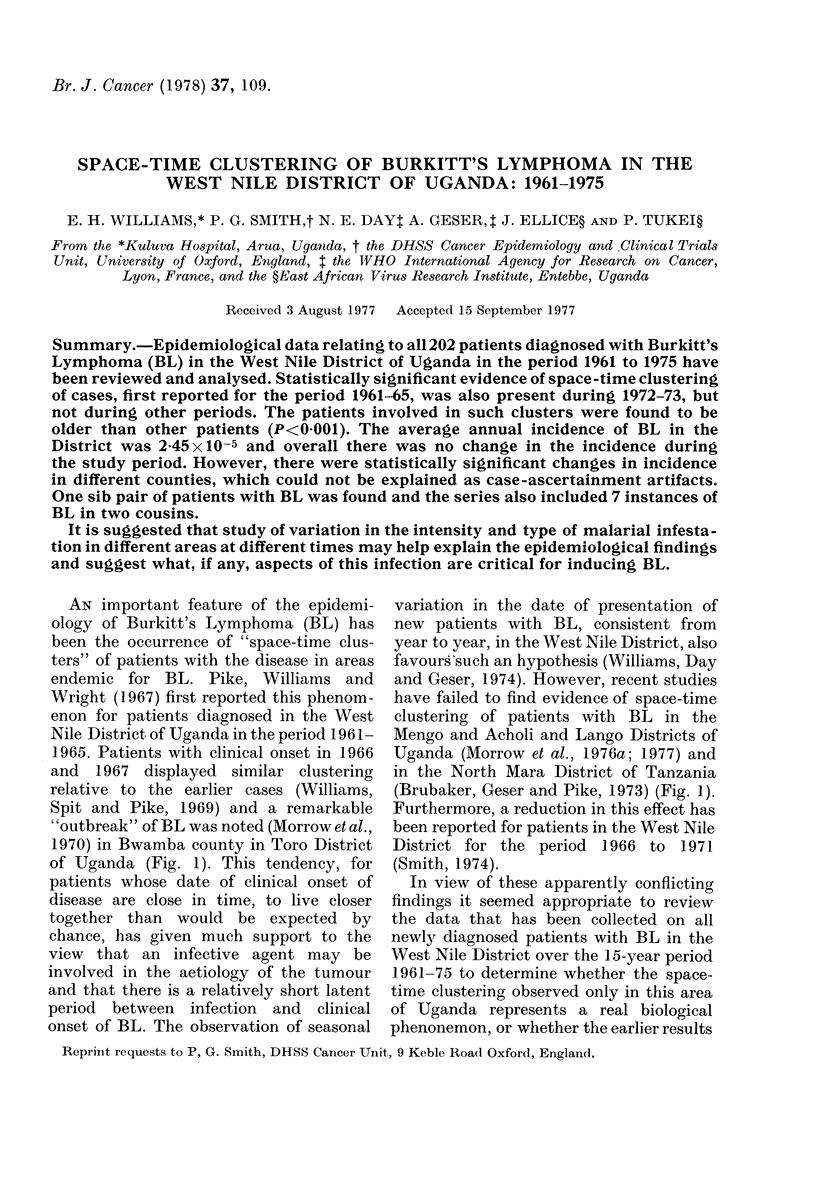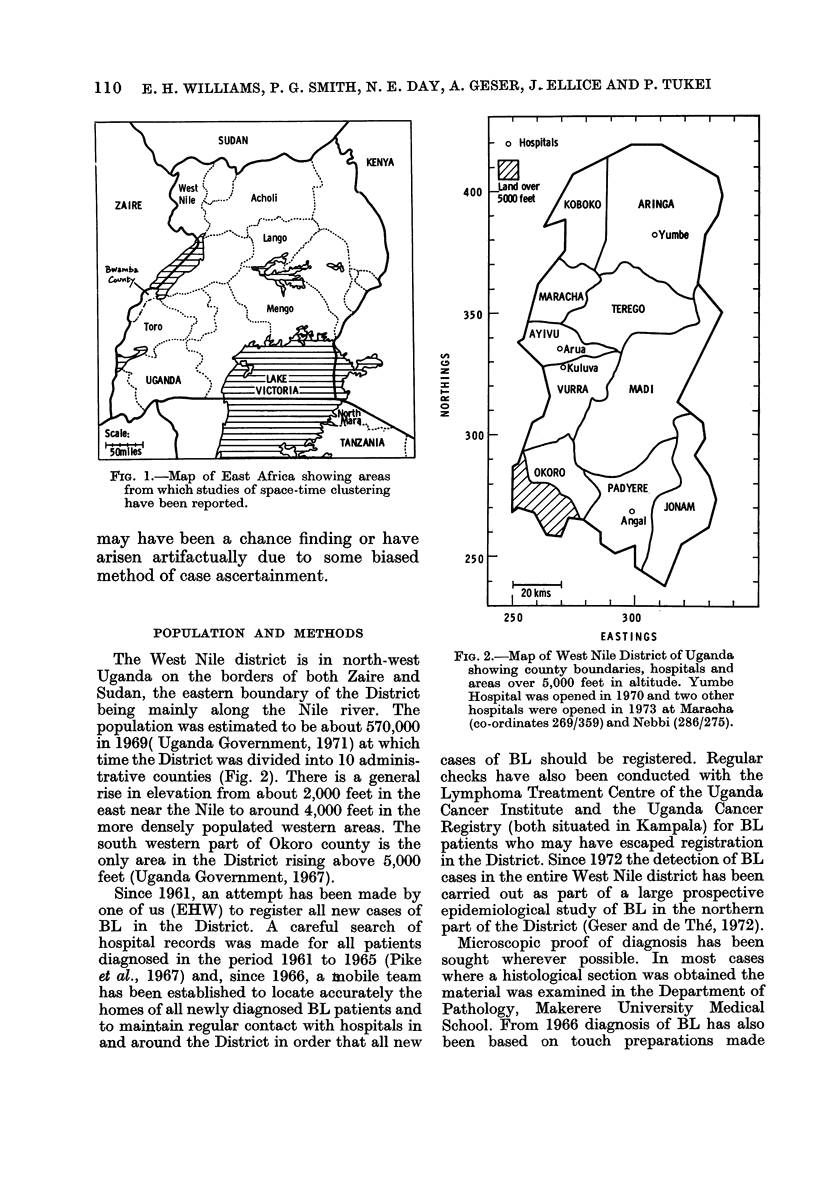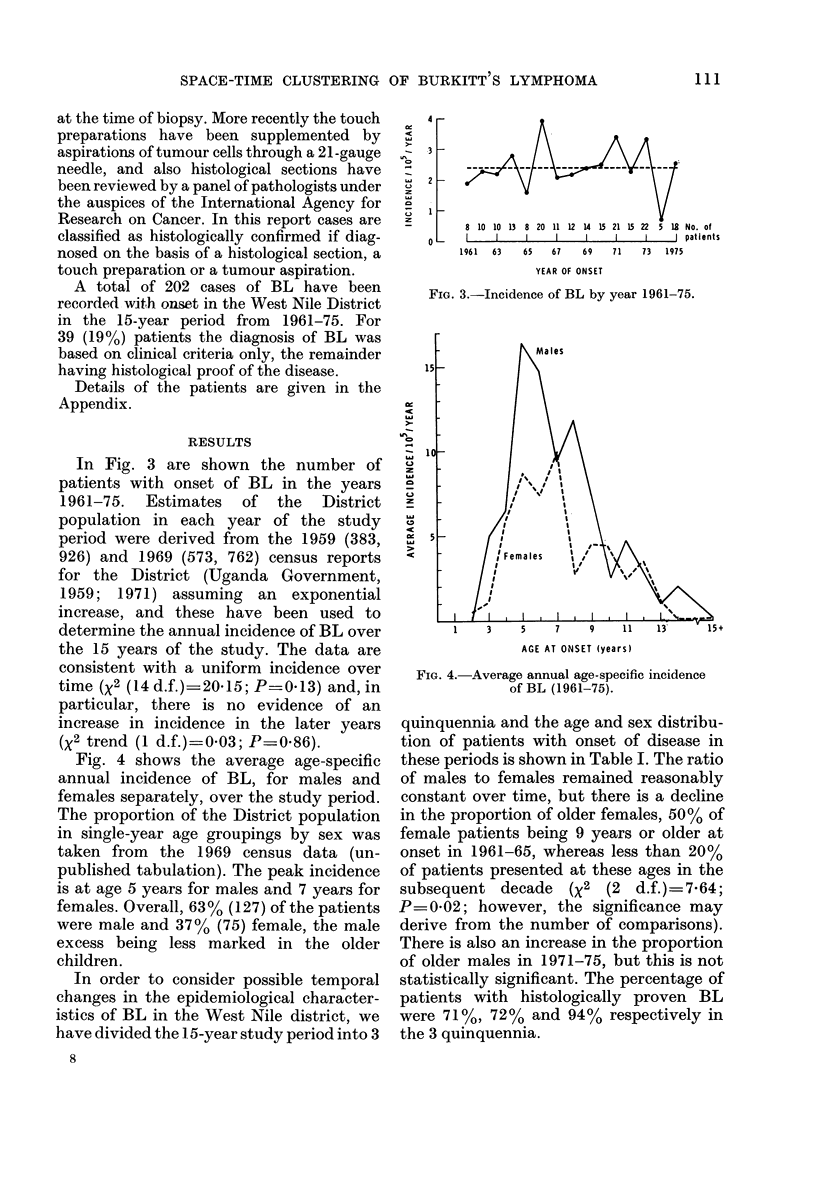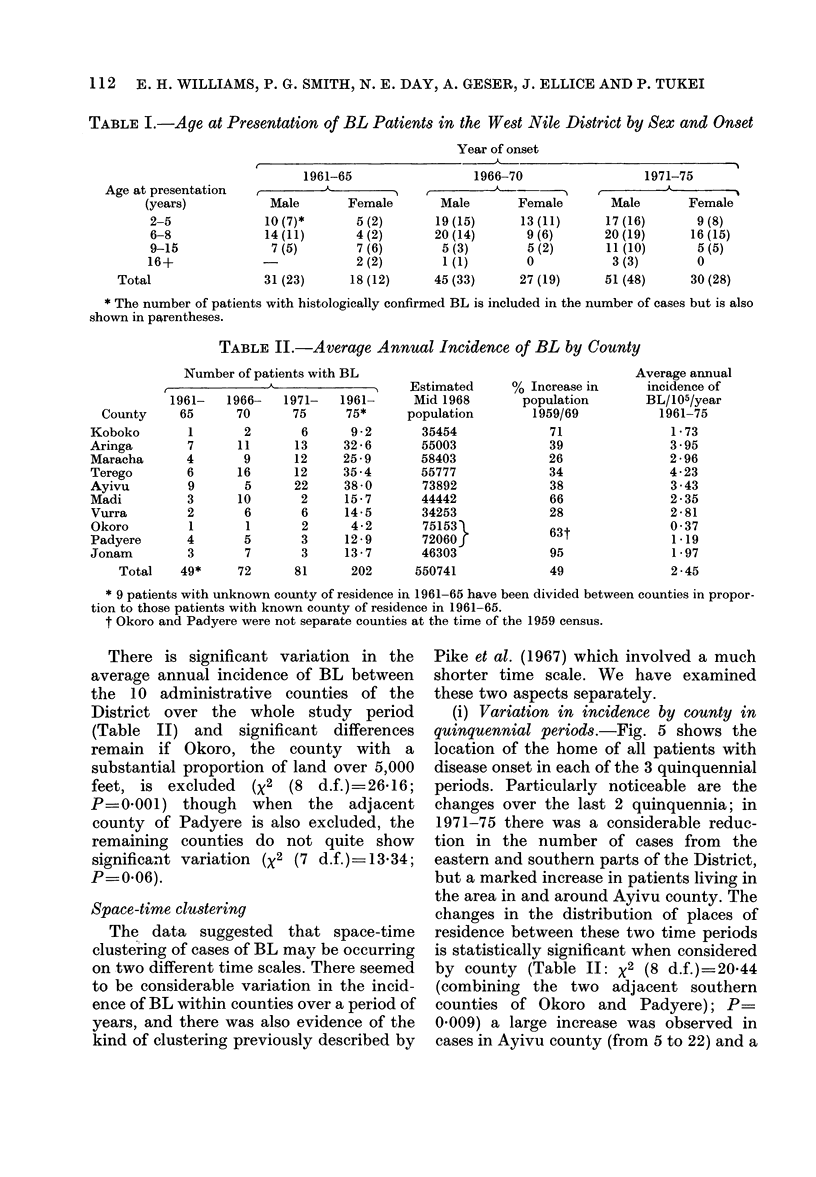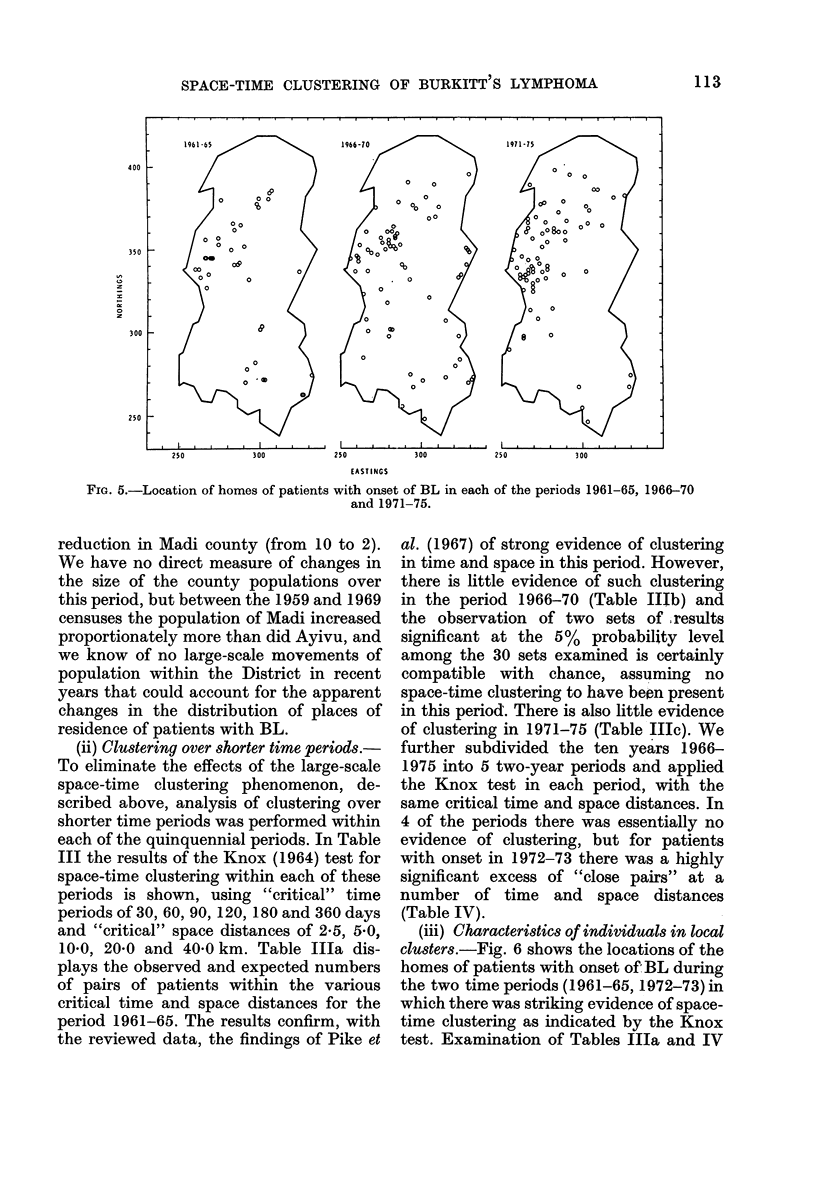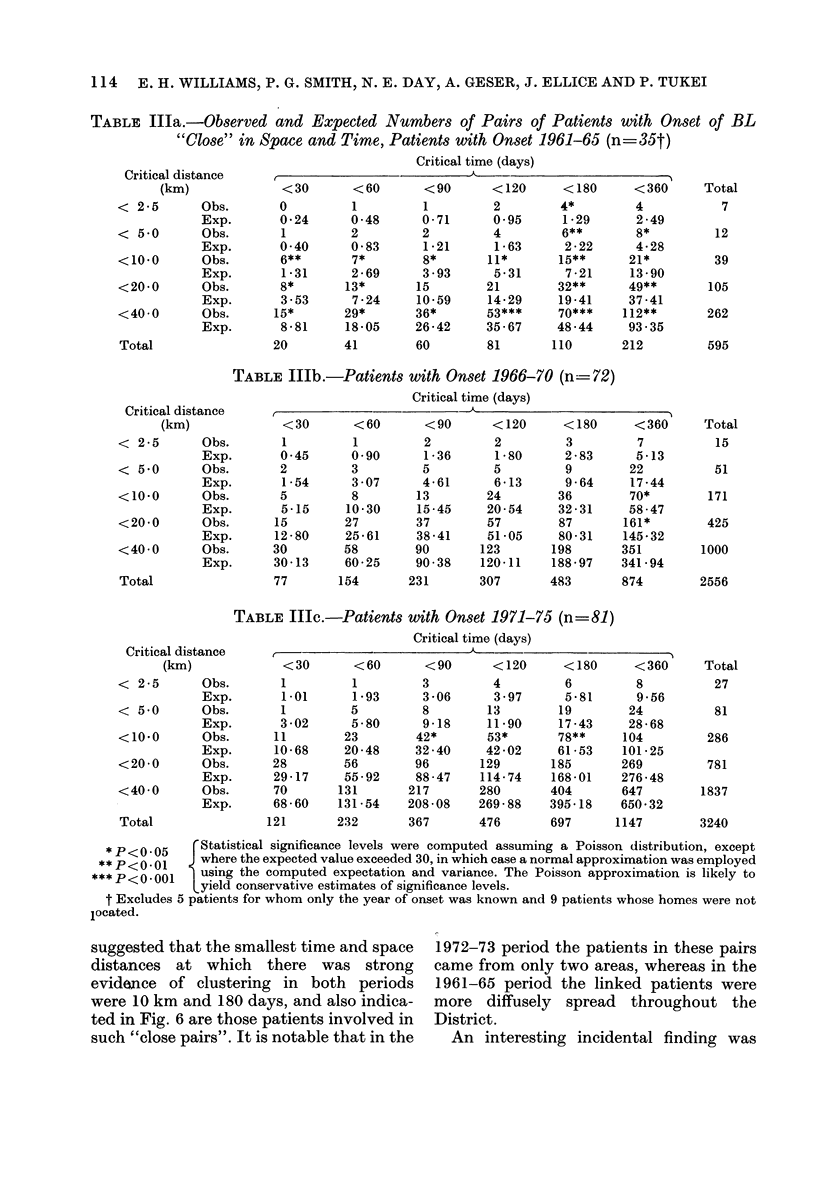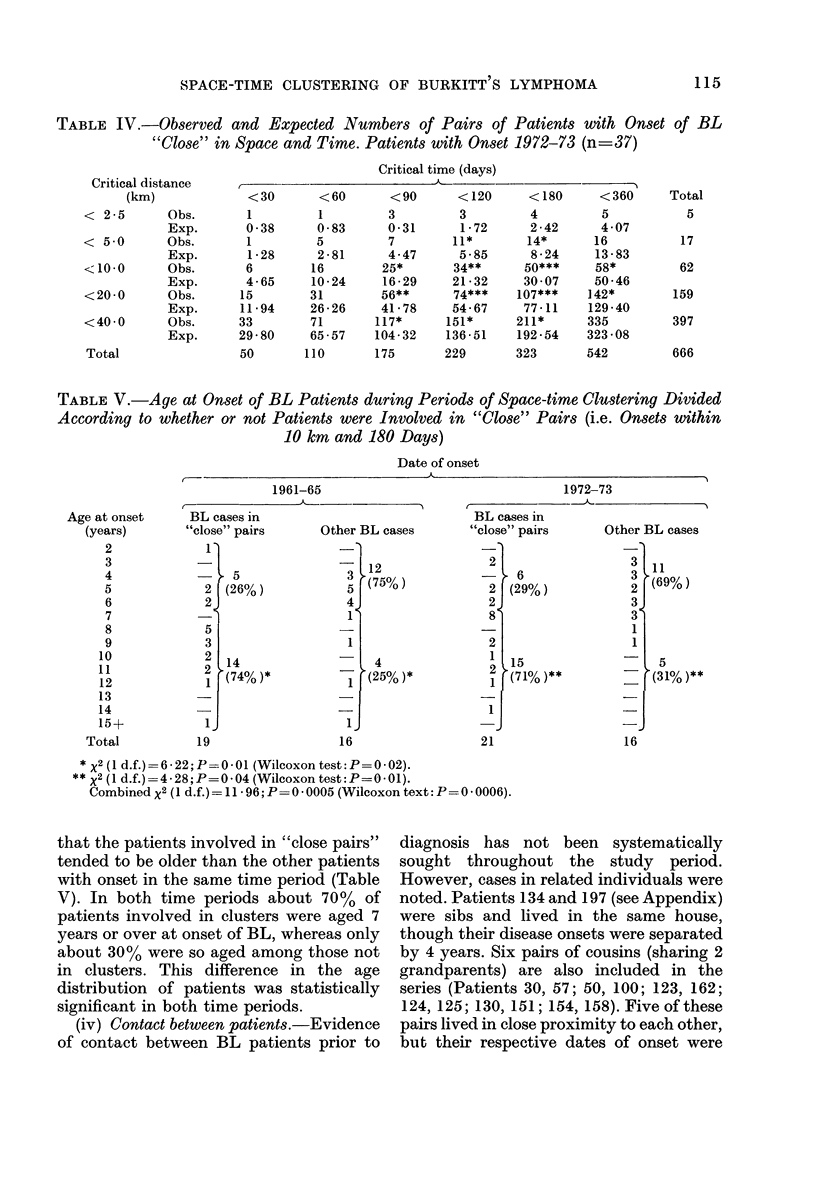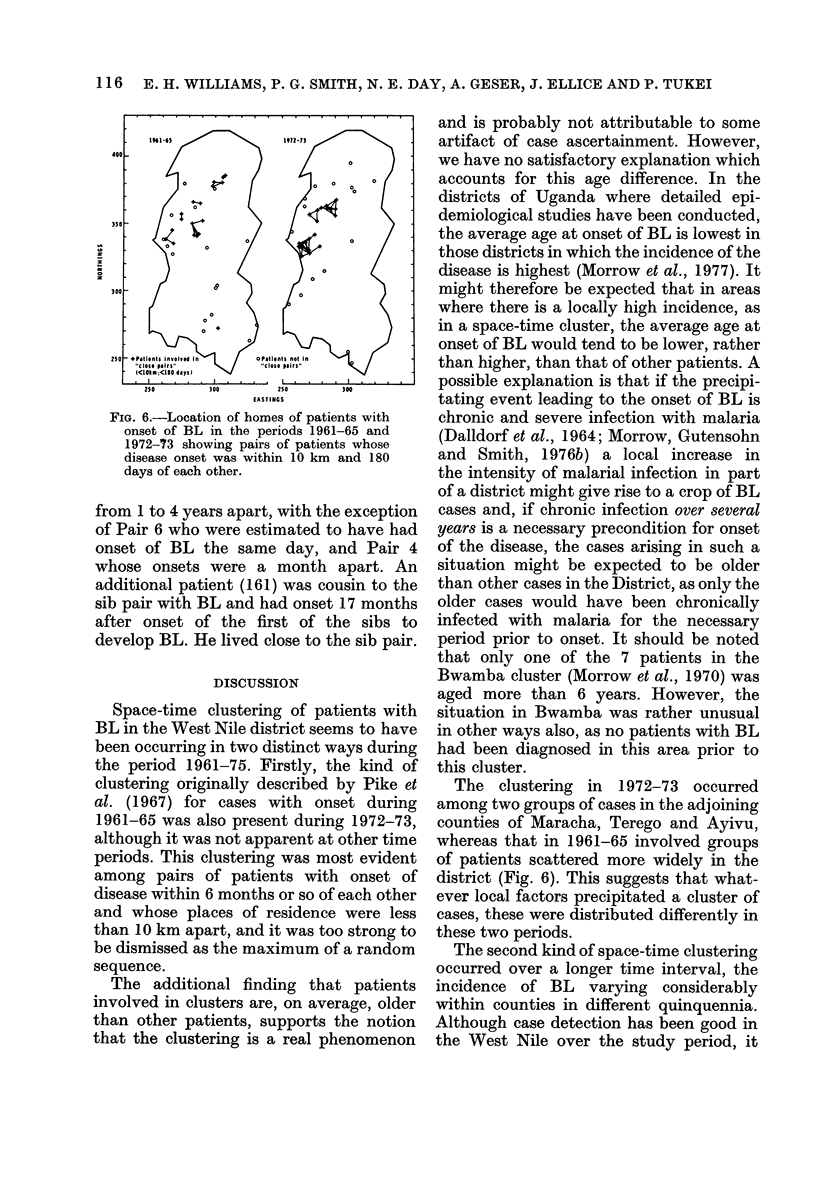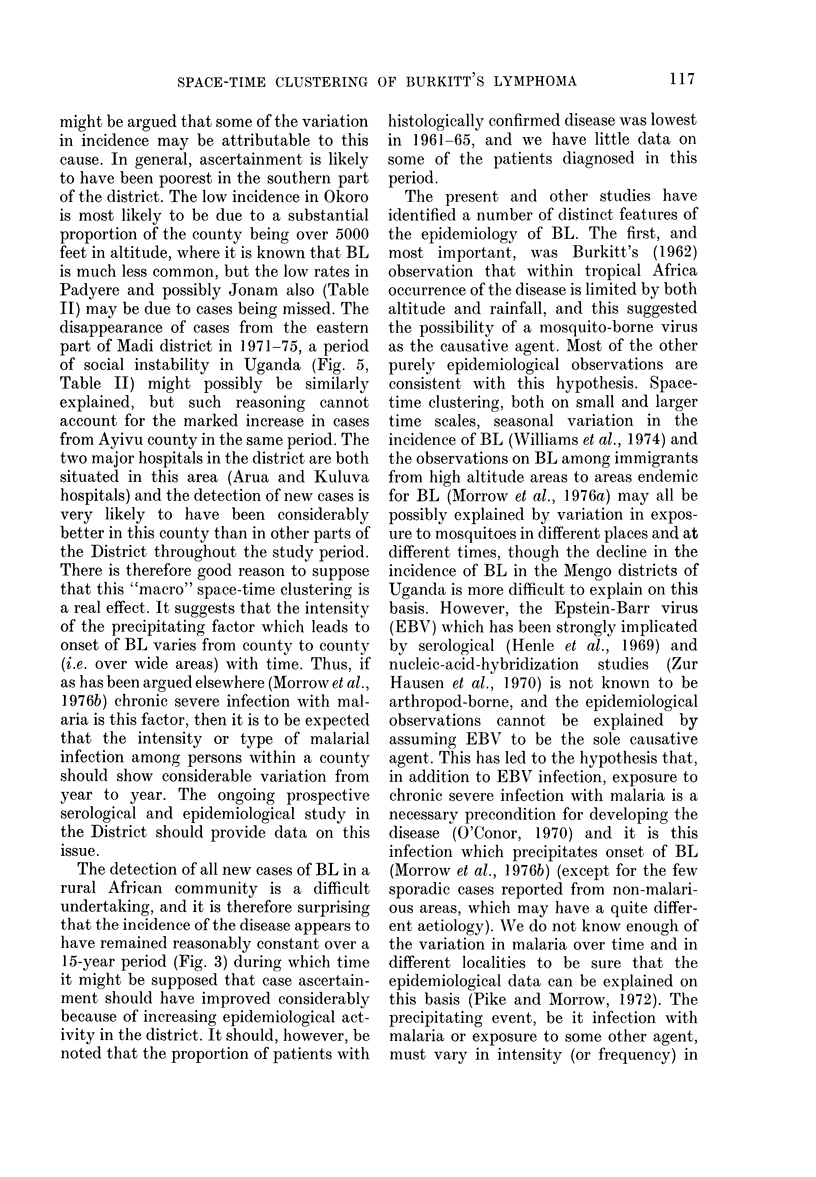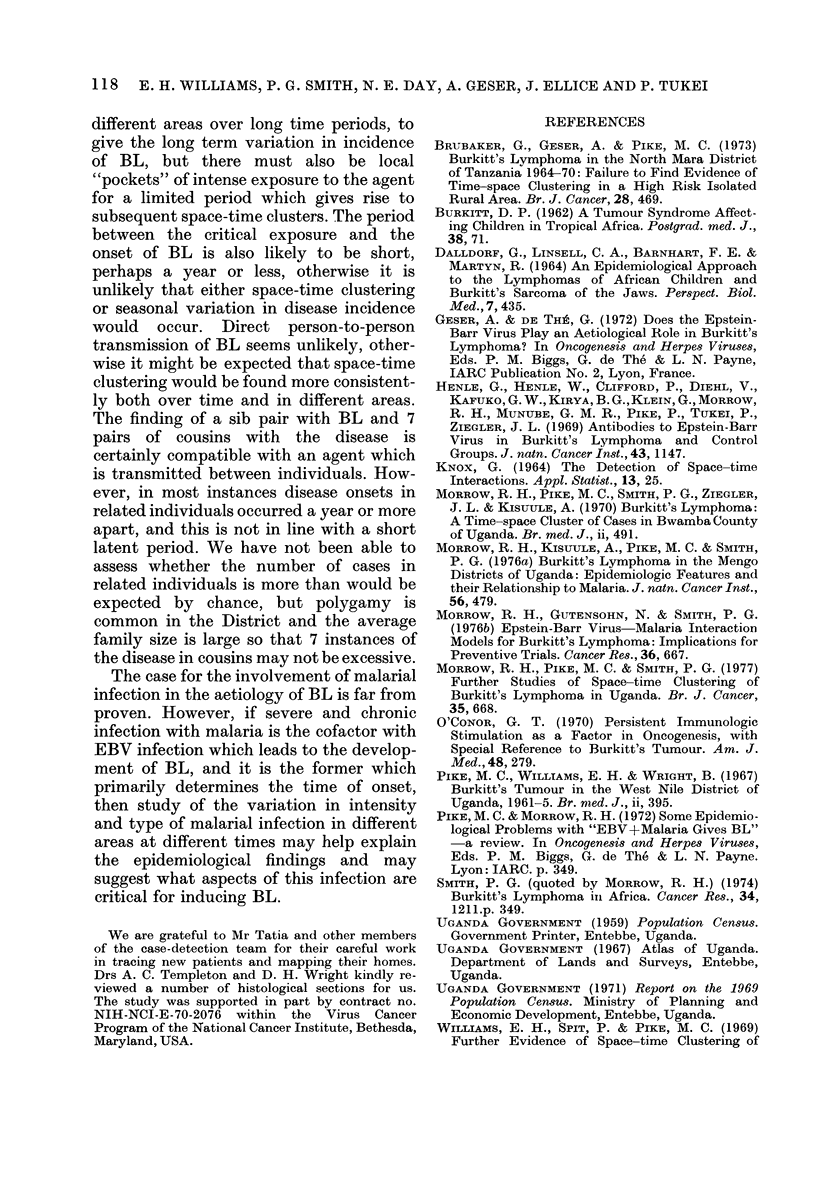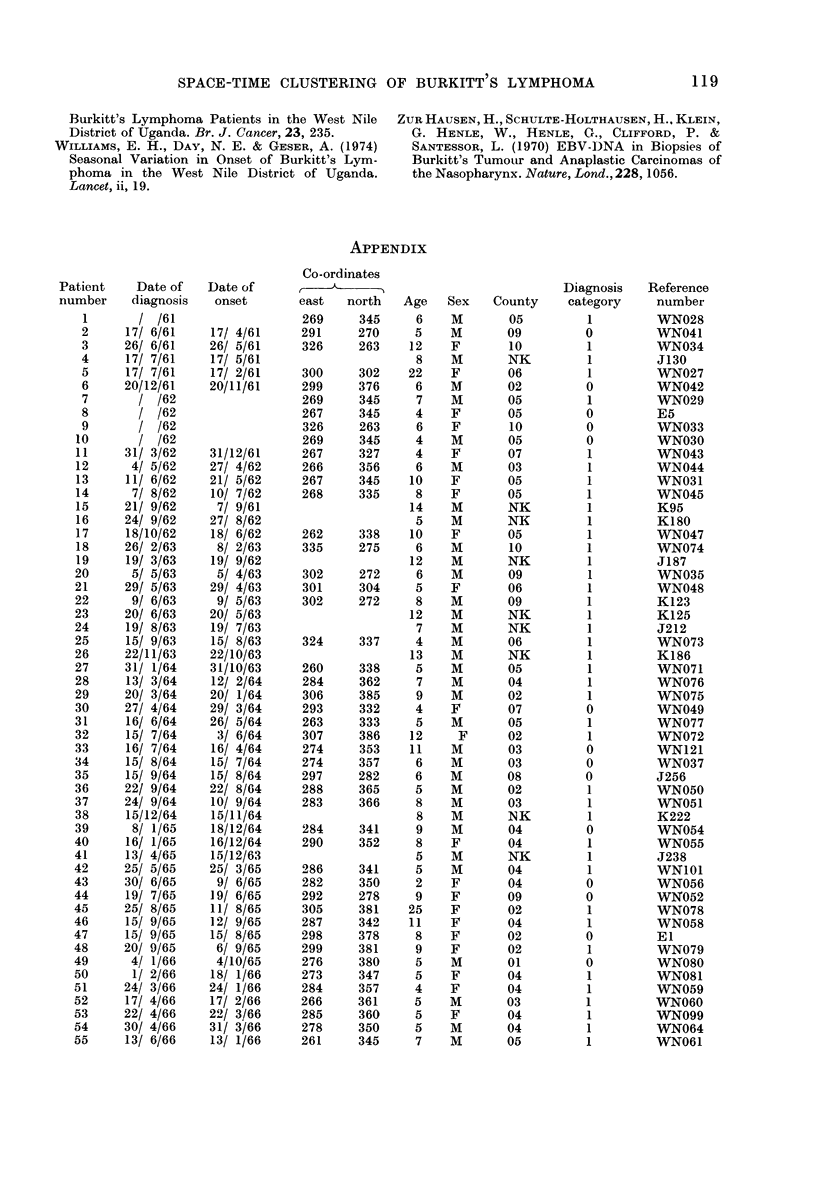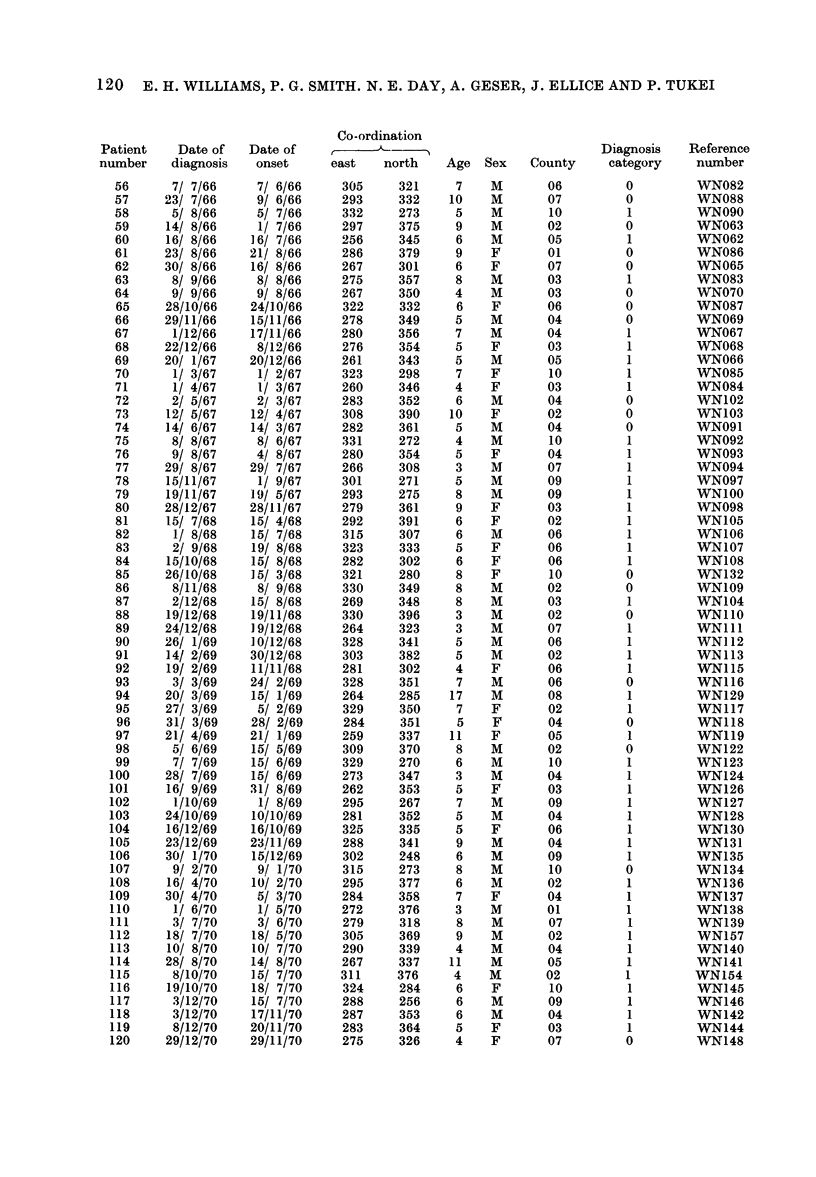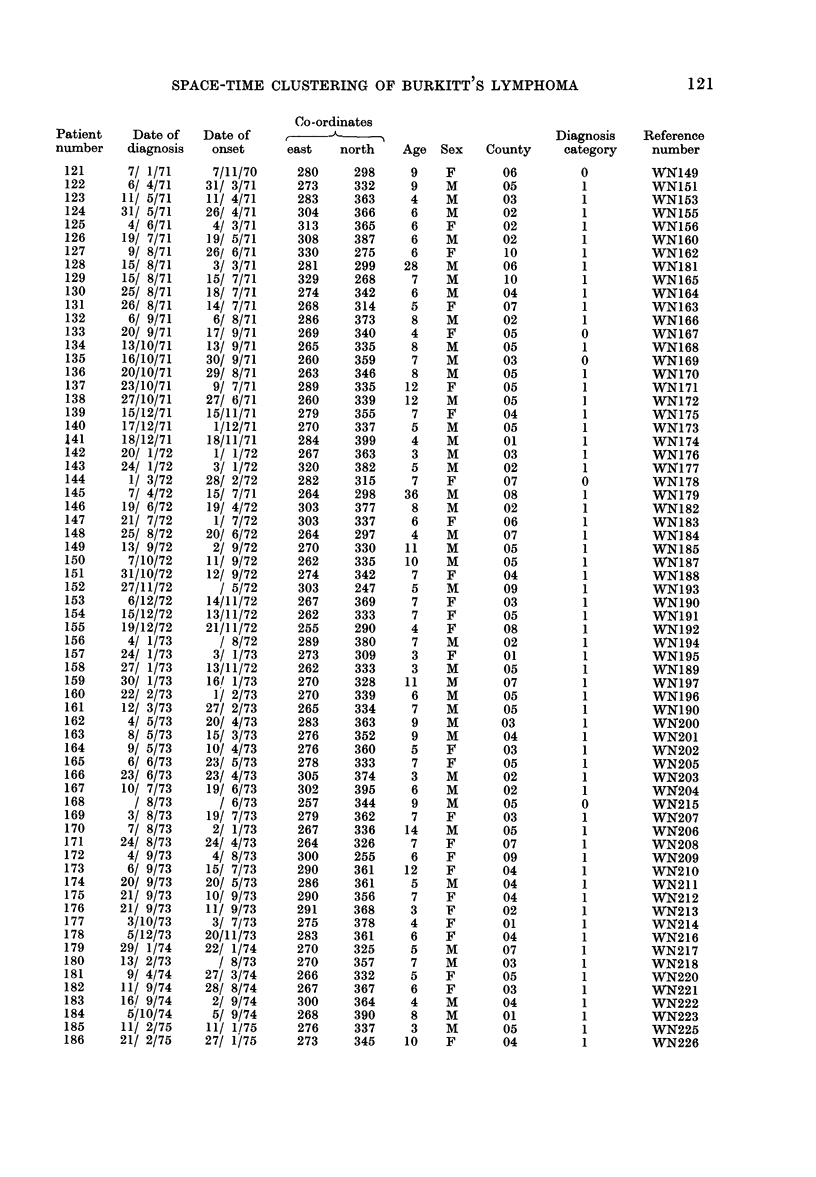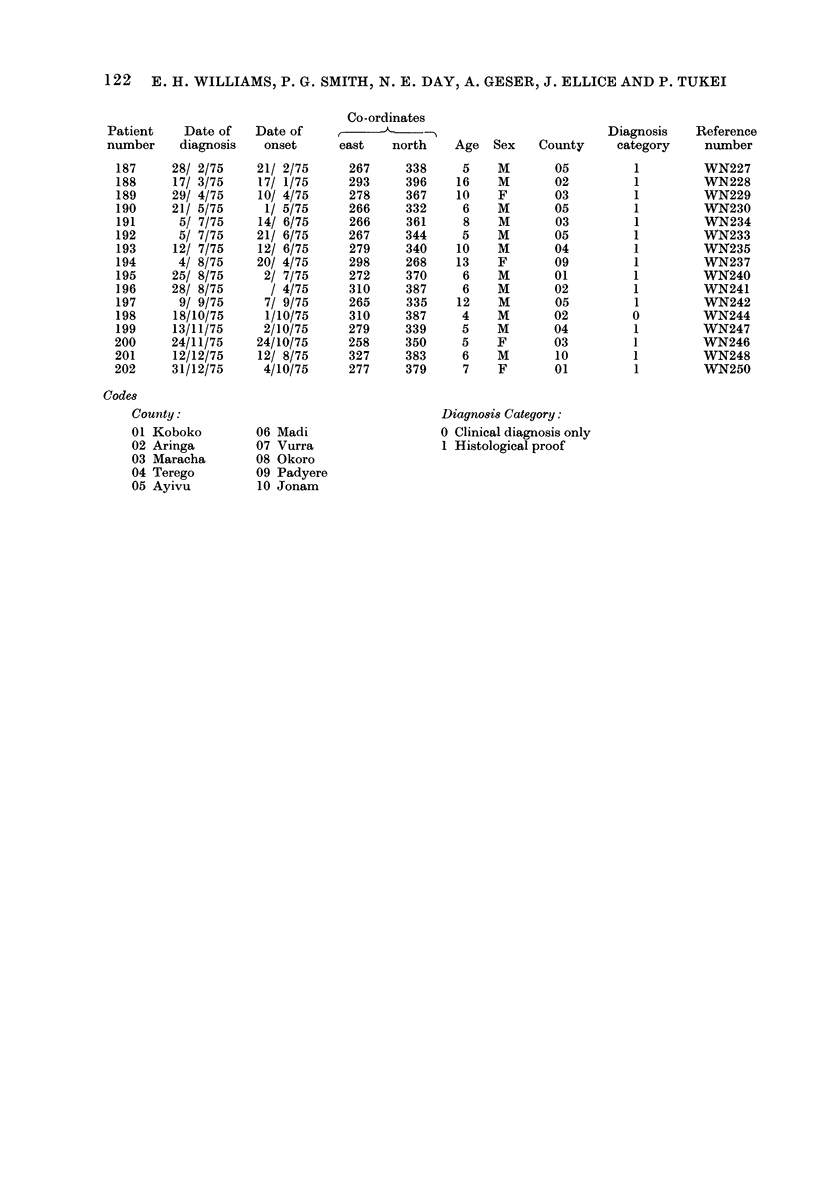# Space-time clustering of Burkitt's lymphoma in the West Nile district of Uganda: 1961-1975.

**DOI:** 10.1038/bjc.1978.16

**Published:** 1978-01

**Authors:** E. H. Williams, P. G. Smith, N. E. Day, A. Geser, J. Ellice, P. Tukei

## Abstract

Epidemiological data relating to all 202 patients diagnosed with Burkitt's Lymphoma (BL) in the West Nile District of Uganda in the period 1961 to 1975 have been reviewed and analysed. Statistically significant evidence of space-time clustering of cases, first reported for the period 1961-65, was also present during 1972-73, but not during other periods. The patients involved in such clusters were found to be older than other patients (P less than 0.001). The average annual incidence of BL in the District was 2.45 x 10(-5) and overall there was no change in the incidence during the study period. However, there were statistically significant changes in incidence in different counties, which could not be explained as case-ascertainment artifacts. One sib pair of patients with BL was found and the series also included 7 instances of BL in two cousins. It is suggested that study of variation in the intensity and type of malarial infestation in different areas at different times may help explain the epidemiological findings and suggest what, if any, aspects of this infection are critical for inducing BL.


*Br. J. Cancer* (1978) **37**, 109.

### SPACE-TIME CLUSTERING OF BURKITT'S LYMPHOMA IN THE WEST NILE DISTRICT OF UGANDA: 1961–1975

E. H. WILLIAMS,* P. G. SMITH,† N. E. DAY‡ A. GESER,‡ J. ELLICE§ AND P. TUKEI§

*From the *Kuluva Hospital, Arua, Uganda, † the DHSS Cancer Epidemiology and Clinical Trials Unit, University of Oxford, England, ‡ the WHO International Agency for Research on Cancer, Lyon, France, and the §East African Virus Research Institute, Entebbe, Uganda*

Received 3 August 1977 Accepted 15 September 1977

**Summary.—Epidemiological data relating to all 202 patients diagnosed with Burkitt's Lymphoma (BL) in the West Nile District of Uganda in the period 1961 to 1975 have been reviewed and analysed. Statistically significant evidence of space-time clustering of cases, first reported for the period 1961–65, was also present during 1972–73, but not during other periods. The patients involved in such clusters were found to be older than other patients ($P<0.001$). The average annual incidence of BL in the District was $2.45\times10^{-5}$ and overall there was no change in the incidence during the study period. However, there were statistically significant changes in incidence in different counties, which could not be explained as case-ascertainment artifacts. One sib pair of patients with BL was found and the series also included 7 instances of BL in two cousins.**

**It is suggested that study of variation in the intensity and type of malarial infestation in different areas at different times may help explain the epidemiological findings and suggest what, if any, aspects of this infection are critical for inducing BL.**

An important feature of the epidemiology of Burkitt's Lymphoma (BL) has been the occurrence of "space-time clusters" of patients with the disease in areas endemic for BL. Pike, Williams and Wright (1967) first reported this phenomenon for patients diagnosed in the West Nile District of Uganda in the period 1961–1965. Patients with clinical onset in 1966 and 1967 displayed similar clustering relative to the earlier cases (Williams, Spit and Pike, 1969) and a remarkable "outbreak" of BL was noted (Morrow *et al.*, 1970) in Bwamba county in Toro District of Uganda (Fig. 1). This tendency, for patients whose date of clinical onset of disease are close in time, to live closer together than would be expected by chance, has given much support to the view that an infective agent may be involved in the aetiology of the tumour and that there is a relatively short latent period between infection and clinical onset of BL. The observation of seasonal variation in the date of presentation of new patients with BL, consistent from year to year, in the West Nile District, also favours such an hypothesis (Williams, Day and Geser, 1974). However, recent studies have failed to find evidence of space-time clustering of patients with BL in the Mengo and Acholi and Lango Districts of Uganda (Morrow *et al.*, 1976*a*; 1977) and in the North Mara District of Tanzania (Brubaker, Geser and Pike, 1973) (Fig. 1). Furthermore, a reduction in this effect has been reported for patients in the West Nile District for the period 1966 to 1971 (Smith, 1974).

In view of these apparently conflicting findings it seemed appropriate to review the data that has been collected on all newly diagnosed patients with BL in the West Nile District over the 15-year period 1961–75 to determine whether the space-time clustering observed only in this area of Uganda represents a real biological phenonemon, or whether the earlier results

Reprint requests to P, G. Smith, DHSS Cancer Unit, 9 Keble Road Oxford, England.

FIG. 1.—Map of East Africa showing areas from which studies of space-time clustering have been reported.

may have been a chance finding or have arisen artifactually due to some biased method of case ascertainment.

### POPULATION AND METHODS

The West Nile district is in north-west Uganda on the borders of both Zaire and Sudan, the eastern boundary of the District being mainly along the Nile river. The population was estimated to be about 570,000 in 1969( Uganda Government, 1971) at which time the District was divided into 10 administrative counties (Fig. 2). There is a general rise in elevation from about 2,000 feet in the east near the Nile to around 4,000 feet in the more densely populated western areas. The south western part of Okoro county is the only area in the District rising above 5,000 feet (Uganda Government, 1967).

Since 1961, an attempt has been made by one of us (EHW) to register all new cases of BL in the District. A careful search of hospital records was made for all patients diagnosed in the period 1961 to 1965 (Pike *et al.*, 1967) and, since 1966, a mobile team has been established to locate accurately the homes of all newly diagnosed BL patients and to maintain regular contact with hospitals in and around the District in order that all new

FIG. 2.—Map of West Nile District of Uganda showing county boundaries, hospitals and areas over 5,000 feet in altitude. Yumbe Hospital was opened in 1970 and two other hospitals were opened in 1973 at Maracha (co-ordinates 269/359) and Nebbi (286/275).

cases of BL should be registered. Regular checks have also been conducted with the Lymphoma Treatment Centre of the Uganda Cancer Institute and the Uganda Cancer Registry (both situated in Kampala) for BL patients who may have escaped registration in the District. Since 1972 the detection of BL cases in the entire West Nile district has been carried out as part of a large prospective epidemiological study of BL in the northern part of the District (Geser and de Thé, 1972).

Microscopic proof of diagnosis has been sought wherever possible. In most cases where a histological section was obtained the material was examined in the Department of Pathology, Makerere University Medical School. From 1966 diagnosis of BL has also been based on touch preparations made at the time of biopsy. More recently the touch preparations have been supplemented by aspirations of tumour cells through a 21-gauge needle, and also histological sections have been reviewed by a panel of pathologists under the auspices of the International Agency for Research on Cancer. In this report cases are classified as histologically confirmed if diagnosed on the basis of a histological section, a touch preparation or a tumour aspiration.

A total of 202 cases of BL have been recorded with onset in the West Nile District in the 15-year period from 1961–75. For 39 (19%) patients the diagnosis of BL was based on clinical criteria only, the remainder having histological proof of the disease.

Details of the patients are given in the Appendix.

### RESULTS

In Fig. 3 are shown the number of patients with onset of BL in the years 1961–75. Estimates of the District population in each year of the study period were derived from the 1959 (383, 926) and 1969 (573, 762) census reports for the District (Uganda Government, 1959; 1971) assuming an exponential increase, and these have been used to determine the annual incidence of BL over the 15 years of the study. The data are consistent with a uniform incidence over time ($\chi^2$ (14 d.f.)=20·15; $P$=0·13) and, in particular, there is no evidence of an increase in incidence in the later years ($\chi^2$ trend (1 d.f.)=0·03; $P$=0·86).

Fig. 4 shows the average age-specific annual incidence of BL, for males and females separately, over the study period. The proportion of the District population in single-year age groupings by sex was taken from the 1969 census data (unpublished tabulation). The peak incidence is at age 5 years for males and 7 years for females. Overall, 63% (127) of the patients were male and 37% (75) female, the male excess being less marked in the older children.

In order to consider possible temporal changes in the epidemiological characteristics of BL in the West Nile district, we have divided the 15-year study period into 3

FIG. 3.—Incidence of BL by year 1961–75.

FIG. 4.—Average annual age-specific incidence of BL (1961–75).

quinquennia and the age and sex distribution of patients with onset of disease in these periods is shown in Table I. The ratio of males to females remained reasonably constant over time, but there is a decline in the proportion of older females, 50% of female patients being 9 years or older at onset in 1961–65, whereas less than 20% of patients presented at these ages in the subsequent decade ($\chi^2$ (2 d.f.)=7·64; $P$=0·02; however, the significance may derive from the number of comparisons). There is also an increase in the proportion of older males in 1971–75, but this is not statistically significant. The percentage of patients with histologically proven BL were 71%, 72% and 94% respectively in the 3 quinquennia.

TABLE I.—*Age at Presentation of BL Patients in the West Nile District by Sex and Onset*

| Age at presentation (years) | Year of onset: 1961–65 Male | 1961–65 Female | 1966–70 Male | 1966–70 Female | 1971–75 Male | 1971–75 Female |
|---|---|---|---|---|---|---|
| 2–5 | 10 (7)* | 5 (2) | 19 (15) | 13 (11) | 17 (16) | 9 (8) |
| 6–8 | 14 (11) | 4 (2) | 20 (14) | 9 (6) | 20 (19) | 16 (15) |
| 9–15 | 7 (5) | 7 (6) | 5 (3) | 5 (2) | 11 (10) | 5 (5) |
| 16+ | — | 2 (2) | 1 (1) | 0 | 3 (3) | 0 |
| Total | 31 (23) | 18 (12) | 45 (33) | 27 (19) | 51 (48) | 30 (28) |

* The number of patients with histologically confirmed BL is included in the number of cases but is also shown in parentheses.

TABLE II.—*Average Annual Incidence of BL by County*

| County | Number of patients with BL: 1961–65 | 1966–70 | 1971–75 | 1961–75* | Estimated Mid 1968 population | % Increase in population 1959/69 | Average annual incidence of BL/$10^5$/year 1961–75 |
|---|---|---|---|---|---|---|---|
| Koboko | 1 | 2 | 6 | 9·2 | 35454 | 71 | 1·73 |
| Aringa | 7 | 11 | 13 | 32·6 | 55003 | 39 | 3·95 |
| Maracha | 4 | 9 | 12 | 25·9 | 58403 | 26 | 2·96 |
| Terego | 6 | 16 | 12 | 35·4 | 55777 | 34 | 4·23 |
| Ayivu | 9 | 5 | 22 | 38·0 | 73892 | 38 | 3·43 |
| Madi | 3 | 10 | 2 | 15·7 | 44442 | 66 | 2·35 |
| Vurra | 2 | 6 | 6 | 14·5 | 34253 | 28 | 2·81 |
| Okoro | 1 | 1 | 2 | 4·2 | 75153 | 63† | 0·37 |
| Padyere | 4 | 5 | 3 | 12·9 | 72060 | | 1·19 |
| Jonam | 3 | 7 | 3 | 13·7 | 46303 | 95 | 1·97 |
| Total | 49* | 72 | 81 | 202 | 550741 | 49 | 2·45 |

* 9 patients with unknown county of residence in 1961–65 have been divided between counties in proportion to those patients with known county of residence in 1961–65.

† Okoro and Padyere were not separate counties at the time of the 1959 census.

There is significant variation in the average annual incidence of BL between the 10 administrative counties of the District over the whole study period (Table II) and significant differences remain if Okoro, the county with a substantial proportion of land over 5,000 feet, is excluded ($\chi^2$ (8 d.f.)=26·16; $P$=0·001) though when the adjacent county of Padyere is also excluded, the remaining counties do not quite show significant variation ($\chi^2$ (7 d.f.)=13·34; $P$=0·06).

### *Space-time clustering*

The data suggested that space-time clustering of cases of BL may be occurring on two different time scales. There seemed to be considerable variation in the incidence of BL within counties over a period of years, and there was also evidence of the kind of clustering previously described by Pike *et al.* (1967) which involved a much shorter time scale. We have examined these two aspects separately.

(i) *Variation in incidence by county in quinquennial periods.*—Fig. 5 shows the location of the home of all patients with disease onset in each of the 3 quinquennial periods. Particularly noticeable are the changes over the last 2 quinquennia; in 1971–75 there was a considerable reduction in the number of cases from the eastern and southern parts of the District, but a marked increase in patients living in the area in and around Ayivu county. The changes in the distribution of places of residence between these two time periods is statistically significant when considered by county (Table II: $\chi^2$ (8 d.f.)=20·44 (combining the two adjacent southern counties of Okoro and Padyere); $P$= 0·009) a large increase was observed in cases in Ayivu county (from 5 to 22) and a

FIG. 5.—Location of homes of patients with onset of BL in each of the periods 1961–65, 1966–70 and 1971–75.

reduction in Madi county (from 10 to 2). We have no direct measure of changes in the size of the county populations over this period, but between the 1959 and 1969 censuses the population of Madi increased proportionately more than did Ayivu, and we know of no large-scale movements of population within the District in recent years that could account for the apparent changes in the distribution of places of residence of patients with BL.

(ii) *Clustering over shorter time periods.*—To eliminate the effects of the large-scale space-time clustering phenomenon, described above, analysis of clustering over shorter time periods was performed within each of the quinquennial periods. In Table III the results of the Knox (1964) test for space-time clustering within each of these periods is shown, using "critical" time periods of 30, 60, 90, 120, 180 and 360 days and "critical" space distances of 2·5, 5·0, 10·0, 20·0 and 40·0 km. Table IIIa displays the observed and expected numbers of pairs of patients within the various critical time and space distances for the period 1961–65. The results confirm, with the reviewed data, the findings of Pike *et al.* (1967) of strong evidence of clustering in time and space in this period. However, there is little evidence of such clustering in the period 1966–70 (Table IIIb) and the observation of two sets of results significant at the 5% probability level among the 30 sets examined is certainly compatible with chance, assuming no space-time clustering to have been present in this period. There is also little evidence of clustering in 1971–75 (Table IIIc). We further subdivided the ten years 1966–1975 into 5 two-year periods and applied the Knox test in each period, with the same critical time and space distances. In 4 of the periods there was essentially no evidence of clustering, but for patients with onset in 1972–73 there was a highly significant excess of "close pairs" at a number of time and space distances (Table IV).

(iii) *Characteristics of individuals in local clusters.*—Fig. 6 shows the locations of the homes of patients with onset of BL during the two time periods (1961–65, 1972–73) in which there was striking evidence of space-time clustering as indicated by the Knox test. Examination of Tables IIIa and IV

TABLE IIIa.—*Observed and Expected Numbers of Pairs of Patients with Onset of BL "Close" in Space and Time, Patients with Onset 1961–65* (n=*35*†)

| Critical distance (km) | | Critical time (days) <30 | <60 | <90 | <120 | <180 | <360 | Total |
|---|---|---|---|---|---|---|---|---|
| < 2·5 | Obs. | 0 | 1 | 1 | 2 | 4* | 4 | 7 |
| | Exp. | 0·24 | 0·48 | 0·71 | 0·95 | 1·29 | 2·49 | |
| < 5·0 | Obs. | 1 | 2 | 2 | 4 | 6** | 8* | 12 |
| | Exp. | 0·40 | 0·83 | 1·21 | 1·63 | 2·22 | 4·28 | |
| <10·0 | Obs. | 6** | 7* | 8* | 11* | 15** | 21* | 39 |
| | Exp. | 1·31 | 2·69 | 3·93 | 5·31 | 7·21 | 13·90 | |
| <20·0 | Obs. | 8* | 13* | 15 | 21 | 32** | 49** | 105 |
| | Exp. | 3·53 | 7·24 | 10·59 | 14·29 | 19·41 | 37·41 | |
| <40·0 | Obs. | 15* | 29* | 36* | 53*** | 70*** | 112** | 262 |
| | Exp. | 8·81 | 18·05 | 26·42 | 35·67 | 48·44 | 93·35 | |
| Total | | 20 | 41 | 60 | 81 | 110 | 212 | 595 |

TABLE IIIb.—*Patients with Onset 1966–70* (n=*72*)

| Critical distance (km) | | Critical time (days) <30 | <60 | <90 | <120 | <180 | <360 | Total |
|---|---|---|---|---|---|---|---|---|
| < 2·5 | Obs. | 1 | 1 | 2 | 2 | 3 | 7 | 15 |
| | Exp. | 0·45 | 0·90 | 1·36 | 1·80 | 2·83 | 5·13 | |
| < 5·0 | Obs. | 2 | 3 | 5 | 5 | 9 | 22 | 51 |
| | Exp. | 1·54 | 3·07 | 4·61 | 6·13 | 9·64 | 17·44 | |
| <10·0 | Obs. | 5 | 8 | 13 | 24 | 36 | 70* | 171 |
| | Exp. | 5·15 | 10·30 | 15·45 | 20·54 | 32·31 | 58·47 | |
| <20·0 | Obs. | 15 | 27 | 37 | 57 | 87 | 161* | 425 |
| | Exp. | 12·80 | 25·61 | 38·41 | 51·05 | 80·31 | 145·32 | |
| <40·0 | Obs. | 30 | 58 | 90 | 123 | 198 | 351 | 1000 |
| | Exp. | 30·13 | 60·25 | 90·38 | 120·11 | 188·97 | 341·94 | |
| Total | | 77 | 154 | 231 | 307 | 483 | 874 | 2556 |

TABLE IIIc.—*Patients with Onset 1971–75* (n=*81*)

| Critical distance (km) | | Critical time (days) <30 | <60 | <90 | <120 | <180 | <360 | Total |
|---|---|---|---|---|---|---|---|---|
| < 2·5 | Obs. | 1 | 1 | 3 | 4 | 6 | 8 | 27 |
| | Exp. | 1·01 | 1·93 | 3·06 | 3·97 | 5·81 | 9·56 | |
| < 5·0 | Obs. | 1 | 5 | 8 | 13 | 19 | 24 | 81 |
| | Exp. | 3·02 | 5·80 | 9·18 | 11·90 | 17·43 | 28·68 | |
| <10·0 | Obs. | 11 | 23 | 42* | 53* | 78** | 104 | 286 |
| | Exp. | 10·68 | 20·48 | 32·40 | 42·02 | 61·53 | 101·25 | |
| <20·0 | Obs. | 28 | 56 | 96 | 129 | 185 | 269 | 781 |
| | Exp. | 29·17 | 55·92 | 88·47 | 114·74 | 168·01 | 276·48 | |
| <40·0 | Obs. | 70 | 131 | 217 | 280 | 404 | 647 | 1837 |
| | Exp. | 68·60 | 131·54 | 208·08 | 269·88 | 395·18 | 650·32 | |
| Total | | 121 | 232 | 367 | 476 | 697 | 1147 | 3240 |

Statistical significance levels were computed assuming a Poisson distribution, except where the expected value exceeded 30, in which case a normal approximation was employed using the computed expectation and variance. The Poisson approximation is likely to yield conservative estimates of significance levels.

† Excludes 5 patients for whom only the year of onset was known and 9 patients whose homes were not located.

suggested that the smallest time and space distances at which there was strong evidence of clustering in both periods were 10 km and 180 days, and also indicated in Fig. 6 are those patients involved in such "close pairs". It is notable that in the 1972–73 period the patients in these pairs came from only two areas, whereas in the 1961–65 period the linked patients were more diffusely spread throughout the District.

An interesting incidental finding was

TABLE IV.—*Observed and Expected Numbers of Pairs of Patients with Onset of BL "Close" in Space and Time. Patients with Onset 1972–73* (n=*37*)

| Critical distance (km) | | Critical time (days) <30 | <60 | <90 | <120 | <180 | <360 | Total |
|---|---|---|---|---|---|---|---|---|
| < 2·5 | Obs. | 1 | 1 | 3 | 3 | 4 | 5 | 5 |
| | Exp. | 0·38 | 0·83 | 0·31 | 1·72 | 2·42 | 4·07 | |
| < 5·0 | Obs. | 1 | 5 | 7 | 11* | 14* | 16 | 17 |
| | Exp. | 1·28 | 2·81 | 4·47 | 5·85 | 8·24 | 13·83 | |
| <10·0 | Obs. | 6 | 16 | 25* | 34** | 50*** | 58* | 62 |
| | Exp. | 4·65 | 10·24 | 16·29 | 21·32 | 30·07 | 50·46 | |
| <20·0 | Obs. | 15 | 31 | 56** | 74*** | 107*** | 142* | 159 |
| | Exp. | 11·94 | 26·26 | 41·78 | 54·67 | 77·11 | 129·40 | |
| <40·0 | Obs. | 33 | 71 | 117* | 151* | 211* | 335 | 397 |
| | Exp. | 29·80 | 65·57 | 104·32 | 136·51 | 192·54 | 323·08 | |
| Total | | 50 | 110 | 175 | 229 | 323 | 542 | 666 |

TABLE V.—*Age at Onset of BL Patients during Periods of Space-time Clustering Divided According to whether or not Patients were Involved in "Close" Pairs* (i.e. *Onsets within 10 km and 180 Days*)

| Age at onset (years) | Date of onset: 1961–65 BL cases in "close" pairs | | 1961–65 Other BL cases | | 1972–73 BL cases in "close" pairs | | 1972–73 Other BL cases | |
|---|---|---|---|---|---|---|---|---|
| 2 | 1 | | — | | — | | — | |
| 3 | — | | — | | 2 | | 3 | |
| 4 | — | 5 (26%) | 3 | 12 (75%) | — | 6 (29%) | 3 | 11 (69%) |
| 5 | 2 | | 5 | | 2 | | 2 | |
| 6 | 2 | | 4 | | 2 | | 3 | |
| 7 | — | | 1 | | 8 | | 3 | |
| 8 | 5 | | — | | — | | 1 | |
| 9 | 3 | | 1 | | 2 | | 1 | |
| 10 | 2 | | — | | 1 | | — | |
| 11 | 2 | 14 (74%)* | — | 4 (25%)* | 2 | 15 (71%)** | — | 5 (31%)** |
| 12 | 1 | | 1 | | 1 | | — | |
| 13 | — | | — | | — | | — | |
| 14 | — | | — | | 1 | | — | |
| 15+ | 1 | | 1 | | — | | — | |
| Total | 19 | | 16 | | 21 | | 16 | |

* $\chi^2$ (1 d.f.) = 6·22; $P$ = 0·01 (Wilcoxon test: $P$ = 0·02).

** $\chi^2$ (1 d.f.) = 4·28; $P$ = 0·04 (Wilcoxon test: $P$ = 0·01).

Combined $\chi^2$ (1 d.f.) = 11·96; $P$ = 0·0005 (Wilcoxon text: $P$ = 0·0006).

that the patients involved in "close pairs" tended to be older than the other patients with onset in the same time period (Table V). In both time periods about 70% of patients involved in clusters were aged 7 years or over at onset of BL, whereas only about 30% were so aged among those not in clusters. This difference in the age distribution of patients was statistically significant in both time periods.

(iv) *Contact between patients.*—Evidence of contact between BL patients prior to diagnosis has not been systematically sought throughout the study period. However, cases in related individuals were noted. Patients 134 and 197 (see Appendix) were sibs and lived in the same house, though their disease onsets were separated by 4 years. Six pairs of cousins (sharing 2 grandparents) are also included in the series (Patients 30, 57; 50, 100; 123, 162; 124, 125; 130, 151; 154, 158). Five of these pairs lived in close proximity to each other, but their respective dates of onset were

FIG. 6.—Location of homes of patients with onset of BL in the periods 1961–65 and 1972–73 showing pairs of patients whose disease onset was within 10 km and 180 days of each other.

from 1 to 4 years apart, with the exception of Pair 6 who were estimated to have had onset of BL the same day, and Pair 4 whose onsets were a month apart. An additional patient (161) was cousin to the sib pair with BL and had onset 17 months after onset of the first of the sibs to develop BL. He lived close to the sib pair.

### DISCUSSION

Space-time clustering of patients with BL in the West Nile district seems to have been occurring in two distinct ways during the period 1961–75. Firstly, the kind of clustering originally described by Pike *et al.* (1967) for cases with onset during 1961–65 was also present during 1972–73, although it was not apparent at other time periods. This clustering was most evident among pairs of patients with onset of disease within 6 months or so of each other and whose places of residence were less than 10 km apart, and it was too strong to be dismissed as the maximum of a random sequence.

The additional finding that patients involved in clusters are, on average, older than other patients, supports the notion that the clustering is a real phenomenon and is probably not attributable to some artifact of case ascertainment. However, we have no satisfactory explanation which accounts for this age difference. In the districts of Uganda where detailed epidemiological studies have been conducted, the average age at onset of BL is lowest in those districts in which the incidence of the disease is highest (Morrow *et al.*, 1977). It might therefore be expected that in areas where there is a locally high incidence, as in a space-time cluster, the average age at onset of BL would tend to be lower, rather than higher, than that of other patients. A possible explanation is that if the precipitating event leading to the onset of BL is chronic and severe infection with malaria (Dalldorf *et al.*, 1964; Morrow, Gutensohn and Smith, 1976*b*) a local increase in the intensity of malarial infection in part of a district might give rise to a crop of BL cases and, if chronic infection *over several years* is a necessary precondition for onset of the disease, the cases arising in such a situation might be expected to be older than other cases in the District, as only the older cases would have been chronically infected with malaria for the necessary period prior to onset. It should be noted that only one of the 7 patients in the Bwamba cluster (Morrow *et al.*, 1970) was aged more than 6 years. However, the situation in Bwamba was rather unusual in other ways also, as no patients with BL had been diagnosed in this area prior to this cluster.

The clustering in 1972–73 occurred among two groups of cases in the adjoining counties of Maracha, Terego and Ayivu, whereas that in 1961–65 involved groups of patients scattered more widely in the district (Fig. 6). This suggests that whatever local factors precipitated a cluster of cases, these were distributed differently in these two periods.

The second kind of space-time clustering occurred over a longer time interval, the incidence of BL varying considerably within counties in different quinquennia. Although case detection has been good in the West Nile over the study period, it might be argued that some of the variation in incidence may be attributable to this cause. In general, ascertainment is likely to have been poorest in the southern part of the district. The low incidence in Okoro is most likely to be due to a substantial proportion of the county being over 5000 feet in altitude, where it is known that BL is much less common, but the low rates in Padyere and possibly Jonam also (Table II) may be due to cases being missed. The disappearance of cases from the eastern part of Madi district in 1971–75, a period of social instability in Uganda (Fig. 5, Table II) might possibly be similarly explained, but such reasoning cannot account for the marked increase in cases from Ayivu county in the same period. The two major hospitals in the district are both situated in this area (Arua and Kuluva hospitals) and the detection of new cases is very likely to have been considerably better in this county than in other parts of the District throughout the study period. There is therefore good reason to suppose that this "macro" space-time clustering is a real effect. It suggests that the intensity of the precipitating factor which leads to onset of BL varies from county to county (*i.e.* over wide areas) with time. Thus, if as has been argued elsewhere (Morrow *et al.*, 1976*b*) chronic severe infection with malaria is this factor, then it is to be expected that the intensity or type of malarial infection among persons within a county should show considerable variation from year to year. The ongoing prospective serological and epidemiological study in the District should provide data on this issue.

The detection of all new cases of BL in a rural African community is a difficult undertaking, and it is therefore surprising that the incidence of the disease appears to have remained reasonably constant over a 15-year period (Fig. 3) during which time it might be supposed that case ascertainment should have improved considerably because of increasing epidemiological activity in the district. It should, however, be noted that the proportion of patients with histologically confirmed disease was lowest in 1961–65, and we have little data on some of the patients diagnosed in this period.

The present and other studies have identified a number of distinct features of the epidemiology of BL. The first, and most important, was Burkitt's (1962) observation that within tropical Africa occurrence of the disease is limited by both altitude and rainfall, and this suggested the possibility of a mosquito-borne virus as the causative agent. Most of the other purely epidemiological observations are consistent with this hypothesis. Space-time clustering, both on small and larger time scales, seasonal variation in the incidence of BL (Williams *et al.*, 1974) and the observations on BL among immigrants from high altitude areas to areas endemic for BL (Morrow *et al.*, 1976*a*) may all be possibly explained by variation in exposure to mosquitoes in different places and at different times, though the decline in the incidence of BL in the Mengo districts of Uganda is more difficult to explain on this basis. However, the Epstein-Barr virus (EBV) which has been strongly implicated by serological (Henle *et al.*, 1969) and nucleic-acid-hybridization studies (Zur Hausen *et al.*, 1970) is not known to be arthropod-borne, and the epidemiological observations cannot be explained by assuming EBV to be the sole causative agent. This has led to the hypothesis that, in addition to EBV infection, exposure to chronic severe infection with malaria is a necessary precondition for developing the disease (O'Conor, 1970) and it is this infection which precipitates onset of BL (Morrow *et al.*, 1976*b*) (except for the few sporadic cases reported from non-malarious areas, which may have a quite different aetiology). We do not know enough of the variation in malaria over time and in different localities to be sure that the epidemiological data can be explained on this basis (Pike and Morrow, 1972). The precipitating event, be it infection with malaria or exposure to some other agent, must vary in intensity (or frequency) in different areas over long time periods, to give the long term variation in incidence of BL, but there must also be local "pockets" of intense exposure to the agent for a limited period which gives rise to subsequent space-time clusters. The period between the critical exposure and the onset of BL is also likely to be short, perhaps a year or less, otherwise it is unlikely that either space-time clustering or seasonal variation in disease incidence would occur. Direct person-to-person transmission of BL seems unlikely, otherwise it might be expected that space-time clustering would be found more consistently both over time and in different areas. The finding of a sib pair with BL and 7 pairs of cousins with the disease is certainly compatible with an agent which is transmitted between individuals. However, in most instances disease onsets in related individuals occurred a year or more apart, and this is not in line with a short latent period. We have not been able to assess whether the number of cases in related individuals is more than would be expected by chance, but polygamy is common in the District and the average family size is large so that 7 instances of the disease in cousins may not be excessive.

The case for the involvement of malarial infection in the aetiology of BL is far from proven. However, if severe and chronic infection with malaria is the cofactor with EBV infection which leads to the development of BL, and it is the former which primarily determines the time of onset, then study of the variation in intensity and type of malarial infection in different areas at different times may help explain the epidemiological findings and may suggest what aspects of this infection are critical for inducing BL.

We are grateful to Mr Tatia and other members of the case-detection team for their careful work in tracing new patients and mapping their homes. Drs A. C. Templeton and D. H. Wright kindly reviewed a number of histological sections for us. The study was supported in part by contract no. NIH-NCI-E-70-2076 within the Virus Cancer Program of the National Cancer Institute, Bethesda, Maryland, USA.

REFERENCES

BRUBAKER, G., GESER, A. & PIKE, M. C. (1973) Burkitt's Lymphoma in the North Mara District of Tanzania 1964–70: Failure to Find Evidence of Time–space Clustering in a High Risk Isolated Rural Area. *Br. J. Cancer*, **28**, 469.

BURKITT, D. P. (1962) A Tumour Syndrome Affecting Children in Tropical Africa. *Postgrad. med. J.*, **38**, 71.

DALLDORF, G., LINSELL, C. A., BARNHART, F. E. & MARTYN, R. (1964) An Epidemiological Approach to the Lymphomas of African Children and Burkitt's Sarcoma of the Jaws. *Perspect. Biol. Med.*, **7**, 435.

GESER, A. & DE THÉ, G. (1972) Does the Epstein-Barr Virus Play an Aetiological Role in Burkitt's Lymphoma? In *Oncogenesis and Herpes Viruses*, Eds. P. M. Biggs, G. de Thé & L. N. Payne, IARC Publication No. 2, Lyon, France.

HENLE, G., HENLE, W., CLIFFORD, P., DIEHL, V., KAFUKO, G. W., KIRYA, B. G., KLEIN, G., MORROW, R. H., MUNUBE, G. M. R., PIKE, P., TUKEI, P., ZIEGLER, J. L. (1969) Antibodies to Epstein-Barr Virus in Burkitt's Lymphoma and Control Groups. *J. natn. Cancer Inst.*, **43**, 1147.

KNOX, G. (1964) The Detection of Space–time Interactions. *Appl. Statist.*, **13**, 25.

MORROW, R. H., PIKE, M. C., SMITH, P. G., ZIEGLER, J. L. & KISUULE, A. (1970) Burkitt's Lymphoma: A Time–space Cluster of Cases in Bwamba County of Uganda. *Br. med. J.*, ii, 491.

MORROW, R. H., KISUULE, A., PIKE, M. C. & SMITH, P. G. (1976*a*) Burkitt's Lymphoma in the Mengo Districts of Uganda: Epidemiologic Features and their Relationship to Malaria. *J. natn. Cancer Inst.*, **56**, 479.

MORROW, R. H., GUTENSOHN, N. & SMITH, P. G. (1976*b*) Epstein-Barr Virus—Malaria Interaction Models for Burkitt's Lymphoma: Implications for Preventive Trials. *Cancer Res.*, **36**, 667.

MORROW, R. H., PIKE, M. C. & SMITH, P. G. (1977) Further Studies of Space–time Clustering of Burkitt's Lymphoma in Uganda. *Br. J. Cancer*, **35**, 668.

O'CONOR, G. T. (1970) Persistent Immunologic Stimulation as a Factor in Oncogenesis, with Special Reference to Burkitt's Tumour. *Am. J. Med.*, **48**, 279.

PIKE, M. C., WILLIAMS, E. H. & WRIGHT, B. (1967) Burkitt's Tumour in the West Nile District of Uganda, 1961–5. *Br. med. J.*, ii, 395.

PIKE, M. C. & MORROW, R. H. (1972) Some Epidemiological Problems with "EBV+Malaria Gives BL" —a review. In *Oncogenesis and Herpes Viruses*, Eds. P. M. Biggs, G. de Thé & L. N. Payne. Lyon: IARC. p. 349.

SMITH, P. G. (quoted by MORROW, R. H.) (1974) Burkitt's Lymphoma in Africa. *Cancer Res.*, **34**, 1211.p. 349.

UGANDA GOVERNMENT (1959) *Population Census*. Government Printer, Entebbe, Uganda.

UGANDA GOVERNMENT (1967) Atlas of Uganda. Department of Lands and Surveys, Entebbe, Uganda.

UGANDA GOVERNMENT (1971) *Report on the 1969 Population Census*. Ministry of Planning and Economic Development, Entebbe, Uganda.

WILLIAMS, E. H., SPIT, P. & PIKE, M. C. (1969) Further Evidence of Space–time Clustering of

Burkitt's Lymphoma Patients in the West Nile District of Uganda. *Br. J. Cancer*, **23**, 235.

WILLIAMS, E. H., DAY, N. E. & GESER, A. (1974) Seasonal Variation in Onset of Burkitt's Lymphoma in the West Nile District of Uganda. *Lancet*, ii, 19.

ZUR HAUSEN, H., SCHULTE-HOLTHAUSEN, H., KLEIN, G. HENLE, W., HENLE, G., CLIFFORD, P. & SANTESSOR, L. (1970) EBV-DNA in Biopsies of Burkitt's Tumour and Anaplastic Carcinomas of the Nasopharynx. *Nature, Lond.*, **228**, 1056.

### APPENDIX

| Patient number | Date of diagnosis | Date of onset | Co-ordinates east | Co-ordinates north | Age | Sex | County | Diagnosis category | Reference number |
|---|---|---|---|---|---|---|---|---|---|
| 1 | / /61 | | 269 | 345 | 6 | M | 05 | 1 | WN028 |
| 2 | 17/ 6/61 | 17/ 4/61 | 291 | 270 | 5 | M | 09 | 0 | WN041 |
| 3 | 26/ 6/61 | 26/ 5/61 | 326 | 263 | 12 | F | 10 | 1 | WN034 |
| 4 | 17/ 7/61 | 17/ 5/61 | | | 8 | M | NK | 1 | J130 |
| 5 | 17/ 7/61 | 17/ 2/61 | 300 | 302 | 22 | F | 06 | 1 | WN027 |
| 6 | 20/12/61 | 20/11/61 | 299 | 376 | 6 | M | 02 | 0 | WN042 |
| 7 | / /62 | | 269 | 345 | 7 | M | 05 | 1 | WN029 |
| 8 | / /62 | | 267 | 345 | 4 | F | 05 | 0 | E5 |
| 9 | / /62 | | 326 | 263 | 6 | F | 10 | 0 | WN033 |
| 10 | / /62 | | 269 | 345 | 4 | M | 05 | 0 | WN030 |
| 11 | 31/ 3/62 | 31/12/61 | 267 | 327 | 4 | F | 07 | 1 | WN043 |
| 12 | 4/ 5/62 | 27/ 4/62 | 266 | 356 | 6 | M | 03 | 1 | WN044 |
| 13 | 11/ 6/62 | 21/ 5/62 | 267 | 345 | 10 | F | 05 | 1 | WN031 |
| 14 | 7/ 8/62 | 10/ 7/62 | 268 | 335 | 8 | F | 05 | 1 | WN045 |
| 15 | 21/ 9/62 | 7/ 9/61 | | | 14 | M | NK | 1 | K95 |
| 16 | 24/ 9/62 | 27/ 8/62 | | | 5 | M | NK | 1 | K180 |
| 17 | 18/10/62 | 18/ 6/62 | 262 | 338 | 10 | F | 05 | 1 | WN047 |
| 18 | 26/ 2/63 | 8/ 2/63 | 335 | 275 | 6 | M | 10 | 1 | WN074 |
| 19 | 19/ 3/63 | 19/ 9/62 | | | 12 | M | NK | 1 | J187 |
| 20 | 5/ 5/63 | 5/ 4/63 | 302 | 272 | 6 | M | 09 | 1 | WN035 |
| 21 | 29/ 5/63 | 29/ 4/63 | 301 | 304 | 5 | F | 06 | 1 | WN048 |
| 22 | 9/ 6/63 | 9/ 5/63 | 302 | 272 | 8 | M | 09 | 1 | K123 |
| 23 | 20/ 6/63 | 20/ 5/63 | | | 12 | M | NK | 1 | K125 |
| 24 | 19/ 8/63 | 19/ 7/63 | | | 7 | M | NK | 1 | J212 |
| 25 | 15/ 9/63 | 15/ 8/63 | 324 | 337 | 4 | M | 06 | 1 | WN073 |
| 26 | 22/11/63 | 22/10/63 | | | 13 | M | NK | 1 | K186 |
| 27 | 31/ 1/64 | 31/10/63 | 260 | 338 | 5 | M | 05 | 1 | WN071 |
| 28 | 13/ 3/64 | 12/ 2/64 | 284 | 362 | 7 | M | 04 | 1 | WN076 |
| 29 | 20/ 3/64 | 20/ 1/64 | 306 | 385 | 9 | M | 02 | 1 | WN075 |
| 30 | 27/ 4/64 | 29/ 3/64 | 293 | 332 | 4 | F | 07 | 0 | WN049 |
| 31 | 16/ 6/64 | 26/ 5/64 | 263 | 333 | 5 | M | 05 | 1 | WN077 |
| 32 | 15/ 7/64 | 3/ 6/64 | 307 | 386 | 12 | F | 02 | 1 | WN072 |
| 33 | 16/ 7/64 | 16/ 4/64 | 274 | 353 | 11 | M | 03 | 0 | WN121 |
| 34 | 15/ 8/64 | 15/ 7/64 | 274 | 357 | 6 | M | 03 | 0 | WN037 |
| 35 | 15/ 9/64 | 15/ 8/64 | 297 | 282 | 6 | M | 08 | 0 | J256 |
| 36 | 22/ 9/64 | 22/ 8/64 | 288 | 365 | 5 | M | 02 | 1 | WN050 |
| 37 | 24/ 9/64 | 10/ 9/64 | 283 | 366 | 8 | M | 03 | 1 | WN051 |
| 38 | 15/12/64 | 15/11/64 | | | 8 | M | NK | 1 | K222 |
| 39 | 8/ 1/65 | 18/12/64 | 284 | 341 | 9 | M | 04 | 0 | WN054 |
| 40 | 16/ 1/65 | 16/12/64 | 290 | 352 | 8 | F | 04 | 1 | WN055 |
| 41 | 13/ 4/65 | 15/12/63 | | | 5 | M | NK | 1 | J238 |
| 42 | 25/ 5/65 | 25/ 3/65 | 286 | 341 | 5 | M | 04 | 1 | WN101 |
| 43 | 30/ 6/65 | 9/ 6/65 | 282 | 350 | 2 | F | 04 | 0 | WN056 |
| 44 | 19/ 7/65 | 19/ 6/65 | 292 | 278 | 9 | F | 09 | 0 | WN052 |
| 45 | 25/ 8/65 | 11/ 8/65 | 305 | 381 | 25 | F | 02 | 1 | WN078 |
| 46 | 15/ 9/65 | 12/ 9/65 | 287 | 342 | 11 | F | 04 | 1 | WN058 |
| 47 | 15/ 9/65 | 15/ 8/65 | 298 | 378 | 8 | F | 02 | 0 | E1 |
| 48 | 20/ 9/65 | 6/ 9/65 | 299 | 381 | 9 | F | 02 | 1 | WN079 |
| 49 | 4/ 1/66 | 4/10/65 | 276 | 380 | 5 | M | 01 | 0 | WN080 |
| 50 | 1/ 2/66 | 18/ 1/66 | 273 | 347 | 5 | F | 04 | 1 | WN081 |
| 51 | 24/ 3/66 | 24/ 1/66 | 284 | 357 | 4 | F | 04 | 1 | WN059 |
| 52 | 17/ 4/66 | 17/ 2/66 | 266 | 361 | 5 | M | 03 | 1 | WN060 |
| 53 | 22/ 4/66 | 22/ 3/66 | 285 | 360 | 5 | F | 04 | 1 | WN099 |
| 54 | 30/ 4/66 | 31/ 3/66 | 278 | 350 | 5 | M | 04 | 1 | WN064 |
| 55 | 13/ 6/66 | 13/ 1/66 | 261 | 345 | 7 | M | 05 | 1 | WN061 |

| Patient number | Date of diagnosis | Date of onset | Co-ordination east | Co-ordination north | Age | Sex | County | Diagnosis category | Reference number |
|---|---|---|---|---|---|---|---|---|---|
| 56 | 7/ 7/66 | 7/ 6/66 | 305 | 321 | 7 | M | 06 | 0 | WN082 |
| 57 | 23/ 7/66 | 9/ 6/66 | 293 | 332 | 10 | M | 07 | 0 | WN088 |
| 58 | 5/ 8/66 | 5/ 7/66 | 332 | 273 | 5 | M | 10 | 1 | WN090 |
| 59 | 14/ 8/66 | 1/ 7/66 | 297 | 375 | 9 | M | 02 | 0 | WN063 |
| 60 | 16/ 8/66 | 16/ 7/66 | 256 | 345 | 6 | M | 05 | 1 | WN062 |
| 61 | 23/ 8/66 | 21/ 8/66 | 286 | 379 | 9 | F | 01 | 0 | WN086 |
| 62 | 30/ 8/66 | 16/ 8/66 | 267 | 301 | 6 | F | 07 | 0 | WN065 |
| 63 | 8/ 9/66 | 8/ 8/66 | 275 | 357 | 8 | M | 03 | 1 | WN083 |
| 64 | 9/ 9/66 | 9/ 8/66 | 267 | 350 | 4 | M | 03 | 0 | WN070 |
| 65 | 28/10/66 | 24/10/66 | 322 | 332 | 6 | F | 06 | 0 | WN087 |
| 66 | 29/11/66 | 15/11/66 | 278 | 349 | 5 | M | 04 | 0 | WN069 |
| 67 | 1/12/66 | 17/11/66 | 280 | 356 | 7 | M | 04 | 1 | WN067 |
| 68 | 22/12/66 | 8/12/66 | 276 | 354 | 5 | F | 03 | 1 | WN068 |
| 69 | 20/ 1/67 | 20/12/66 | 261 | 343 | 5 | M | 05 | 1 | WN066 |
| 70 | 1/ 3/67 | 1/ 2/67 | 323 | 298 | 7 | F | 10 | 1 | WN085 |
| 71 | 1/ 4/67 | 1/ 3/67 | 260 | 346 | 4 | F | 03 | 1 | WN084 |
| 72 | 2/ 5/67 | 2/ 3/67 | 283 | 352 | 6 | M | 04 | 0 | WN102 |
| 73 | 12/ 5/67 | 12/ 4/67 | 308 | 390 | 10 | F | 02 | 0 | WN103 |
| 74 | 14/ 6/67 | 14/ 3/67 | 282 | 361 | 5 | M | 04 | 0 | WN091 |
| 75 | 8/ 8/67 | 8/ 6/67 | 331 | 272 | 4 | M | 10 | 1 | WN092 |
| 76 | 9/ 8/67 | 4/ 8/67 | 280 | 354 | 5 | F | 04 | 1 | WN093 |
| 77 | 29/ 8/67 | 29/ 7/67 | 266 | 308 | 3 | M | 07 | 1 | WN094 |
| 78 | 15/11/67 | 1/ 9/67 | 301 | 271 | 5 | M | 09 | 1 | WN097 |
| 79 | 19/11/67 | 19/ 5/67 | 293 | 275 | 8 | M | 09 | 1 | WN100 |
| 80 | 28/12/67 | 28/11/67 | 279 | 361 | 9 | F | 03 | 1 | WN098 |
| 81 | 15/ 7/68 | 15/ 4/68 | 292 | 391 | 6 | F | 02 | 1 | WN105 |
| 82 | 1/ 8/68 | 15/ 7/68 | 315 | 307 | 6 | M | 06 | 1 | WN106 |
| 83 | 2/ 9/68 | 19/ 8/68 | 323 | 333 | 5 | F | 06 | 1 | WN107 |
| 84 | 15/10/68 | 15/ 8/68 | 282 | 302 | 6 | F | 06 | 1 | WN108 |
| 85 | 26/10/68 | 15/ 3/68 | 321 | 280 | 8 | F | 10 | 0 | WN132 |
| 86 | 8/11/68 | 8/ 9/68 | 330 | 349 | 8 | M | 02 | 0 | WN109 |
| 87 | 2/12/68 | 15/ 8/68 | 269 | 348 | 8 | M | 03 | 1 | WN104 |
| 88 | 19/12/68 | 19/11/68 | 330 | 396 | 3 | M | 02 | 0 | WN110 |
| 89 | 24/12/68 | 19/12/68 | 264 | 323 | 3 | M | 07 | 1 | WN111 |
| 90 | 26/ 1/69 | 10/12/68 | 328 | 341 | 5 | M | 06 | 1 | WN112 |
| 91 | 14/ 2/69 | 30/12/68 | 303 | 382 | 5 | M | 02 | 1 | WN113 |
| 92 | 19/ 2/69 | 11/11/68 | 281 | 302 | 4 | F | 06 | 1 | WN115 |
| 93 | 3/ 3/69 | 24/ 2/69 | 328 | 351 | 7 | M | 06 | 0 | WN116 |
| 94 | 20/ 3/69 | 15/ 1/69 | 264 | 285 | 17 | M | 08 | 1 | WN129 |
| 95 | 27/ 3/69 | 5/ 2/69 | 329 | 350 | 7 | F | 02 | 1 | WN117 |
| 96 | 31/ 3/69 | 28/ 2/69 | 284 | 351 | 5 | F | 04 | 0 | WN118 |
| 97 | 21/ 4/69 | 21/ 1/69 | 259 | 337 | 11 | F | 05 | 1 | WN119 |
| 98 | 5/ 6/69 | 15/ 5/69 | 309 | 370 | 8 | M | 02 | 0 | WN122 |
| 99 | 7/ 7/69 | 15/ 6/69 | 329 | 270 | 6 | M | 10 | 1 | WN123 |
| 100 | 28/ 7/69 | 15/ 6/69 | 273 | 347 | 3 | M | 04 | 1 | WN124 |
| 101 | 16/ 9/69 | 31/ 8/69 | 262 | 353 | 5 | F | 03 | 1 | WN126 |
| 102 | 1/10/69 | 1/ 8/69 | 295 | 267 | 7 | M | 09 | 1 | WN127 |
| 103 | 24/10/69 | 10/10/69 | 281 | 352 | 5 | M | 04 | 1 | WN128 |
| 104 | 16/12/69 | 16/10/69 | 325 | 335 | 5 | F | 06 | 1 | WN130 |
| 105 | 23/12/69 | 23/11/69 | 288 | 341 | 9 | M | 04 | 1 | WN131 |
| 106 | 30/ 1/70 | 15/12/69 | 302 | 248 | 6 | M | 09 | 1 | WN135 |
| 107 | 9/ 2/70 | 9/ 1/70 | 315 | 273 | 8 | M | 10 | 0 | WN134 |
| 108 | 16/ 4/70 | 10/ 2/70 | 295 | 377 | 6 | M | 02 | 1 | WN136 |
| 109 | 30/ 4/70 | 5/ 3/70 | 284 | 358 | 7 | F | 04 | 1 | WN137 |
| 110 | 1/ 6/70 | 1/ 5/70 | 272 | 376 | 3 | M | 01 | 1 | WN138 |
| 111 | 3/ 7/70 | 3/ 6/70 | 279 | 318 | 8 | M | 07 | 1 | WN139 |
| 112 | 18/ 7/70 | 18/ 5/70 | 305 | 369 | 9 | M | 02 | 1 | WN157 |
| 113 | 10/ 8/70 | 10/ 7/70 | 290 | 339 | 4 | M | 04 | 1 | WN140 |
| 114 | 28/ 8/70 | 14/ 8/70 | 267 | 337 | 11 | M | 05 | 1 | WN141 |
| 115 | 8/10/70 | 15/ 7/70 | 311 | 376 | 4 | M | 02 | 1 | WN154 |
| 116 | 19/10/70 | 18/ 7/70 | 324 | 284 | 6 | F | 10 | 1 | WN145 |
| 117 | 3/12/70 | 15/ 7/70 | 288 | 256 | 6 | M | 09 | 1 | WN146 |
| 118 | 3/12/70 | 17/11/70 | 287 | 353 | 6 | M | 04 | 1 | WN142 |
| 119 | 8/12/70 | 20/11/70 | 283 | 364 | 5 | F | 03 | 1 | WN144 |
| 120 | 29/12/70 | 29/11/70 | 275 | 326 | 4 | F | 07 | 0 | WN148 |

| Patient number | Date of diagnosis | Date of onset | Co-ordinates east | Co-ordinates north | Age | Sex | County | Diagnosis category | Reference number |
|---|---|---|---|---|---|---|---|---|---|
| 121 | 7/ 1/71 | 7/11/70 | 280 | 298 | 9 | F | 06 | 0 | WN149 |
| 122 | 6/ 4/71 | 31/ 3/71 | 273 | 332 | 9 | M | 05 | 1 | WN151 |
| 123 | 11/ 5/71 | 11/ 4/71 | 283 | 363 | 4 | M | 03 | 1 | WN153 |
| 124 | 31/ 5/71 | 26/ 4/71 | 304 | 366 | 6 | M | 02 | 1 | WN155 |
| 125 | 4/ 6/71 | 4/ 3/71 | 313 | 365 | 6 | F | 02 | 1 | WN156 |
| 126 | 19/ 7/71 | 19/ 5/71 | 308 | 387 | 6 | M | 02 | 1 | WN160 |
| 127 | 9/ 8/71 | 26/ 6/71 | 330 | 275 | 6 | F | 10 | 1 | WN162 |
| 128 | 15/ 8/71 | 3/ 3/71 | 281 | 299 | 28 | M | 06 | 1 | WN181 |
| 129 | 15/ 8/71 | 15/ 7/71 | 329 | 268 | 7 | M | 10 | 1 | WN165 |
| 130 | 25/ 8/71 | 18/ 7/71 | 274 | 342 | 6 | M | 04 | 1 | WN164 |
| 131 | 26/ 8/71 | 14/ 7/71 | 268 | 314 | 5 | F | 07 | 1 | WN163 |
| 132 | 6/ 9/71 | 6/ 8/71 | 286 | 373 | 8 | M | 02 | 1 | WN166 |
| 133 | 20/ 9/71 | 17/ 9/71 | 269 | 340 | 4 | F | 05 | 0 | WN167 |
| 134 | 13/10/71 | 13/ 9/71 | 265 | 335 | 8 | M | 05 | 1 | WN168 |
| 135 | 16/10/71 | 30/ 9/71 | 260 | 359 | 7 | M | 03 | 0 | WN169 |
| 136 | 20/10/71 | 29/ 8/71 | 263 | 346 | 8 | M | 05 | 1 | WN170 |
| 137 | 23/10/71 | 9/ 7/71 | 289 | 335 | 12 | F | 05 | 1 | WN171 |
| 138 | 27/10/71 | 27/ 6/71 | 260 | 339 | 12 | M | 05 | 1 | WN172 |
| 139 | 15/12/71 | 15/11/71 | 279 | 355 | 7 | F | 04 | 1 | WN175 |
| 140 | 17/12/71 | 1/12/71 | 270 | 337 | 5 | M | 05 | 1 | WN173 |
| 141 | 18/12/71 | 18/11/71 | 284 | 399 | 4 | M | 01 | 1 | WN174 |
| 142 | 20/ 1/72 | 1/ 1/72 | 267 | 363 | 3 | M | 03 | 1 | WN176 |
| 143 | 24/ 1/72 | 3/ 1/72 | 320 | 382 | 5 | M | 02 | 1 | WN177 |
| 144 | 1/ 3/72 | 28/ 2/72 | 282 | 315 | 7 | F | 07 | 0 | WN178 |
| 145 | 7/ 4/72 | 15/ 7/71 | 264 | 298 | 36 | M | 08 | 1 | WN179 |
| 146 | 19/ 6/72 | 19/ 4/72 | 303 | 377 | 8 | M | 02 | 1 | WN182 |
| 147 | 21/ 7/72 | 1/ 7/72 | 303 | 337 | 6 | F | 06 | 1 | WN183 |
| 148 | 25/ 8/72 | 20/ 6/72 | 264 | 297 | 4 | M | 07 | 1 | WN184 |
| 149 | 13/ 9/72 | 2/ 9/72 | 270 | 330 | 11 | M | 05 | 1 | WN185 |
| 150 | 7/10/72 | 11/ 9/72 | 262 | 335 | 10 | M | 05 | 1 | WN187 |
| 151 | 31/10/72 | 12/ 9/72 | 274 | 342 | 7 | F | 04 | 1 | WN188 |
| 152 | 27/11/72 | / 5/72 | 303 | 247 | 5 | M | 09 | 1 | WN193 |
| 153 | 6/12/72 | 14/11/72 | 267 | 369 | 7 | F | 03 | 1 | WN190 |
| 154 | 15/12/72 | 13/11/72 | 262 | 333 | 7 | F | 05 | 1 | WN191 |
| 155 | 19/12/72 | 21/11/72 | 255 | 290 | 4 | F | 08 | 1 | WN192 |
| 156 | 4/ 1/73 | / 8/72 | 289 | 380 | 7 | M | 02 | 1 | WN194 |
| 157 | 24/ 1/73 | 3/ 1/73 | 273 | 309 | 3 | F | 01 | 1 | WN195 |
| 158 | 27/ 1/73 | 13/11/72 | 262 | 333 | 3 | M | 05 | 1 | WN189 |
| 159 | 30/ 1/73 | 16/ 1/73 | 270 | 328 | 11 | M | 07 | 1 | WN197 |
| 160 | 22/ 2/73 | 1/ 2/73 | 270 | 339 | 6 | M | 05 | 1 | WN196 |
| 161 | 12/ 3/73 | 27/ 2/73 | 265 | 334 | 7 | M | 05 | 1 | WN190 |
| 162 | 4/ 5/73 | 20/ 4/73 | 283 | 363 | 9 | M | 03 | 1 | WN200 |
| 163 | 8/ 5/73 | 15/ 3/73 | 276 | 352 | 9 | M | 04 | 1 | WN201 |
| 164 | 9/ 5/73 | 10/ 4/73 | 276 | 360 | 5 | F | 03 | 1 | WN202 |
| 165 | 6/ 6/73 | 23/ 5/73 | 278 | 333 | 7 | F | 05 | 1 | WN205 |
| 166 | 23/ 6/73 | 23/ 4/73 | 305 | 374 | 3 | M | 02 | 1 | WN203 |
| 167 | 10/ 7/73 | 19/ 6/73 | 302 | 395 | 6 | M | 02 | 1 | WN204 |
| 168 | / 8/73 | / 6/73 | 257 | 344 | 9 | M | 05 | 0 | WN215 |
| 169 | 3/ 8/73 | 19/ 7/73 | 279 | 362 | 7 | F | 03 | 1 | WN207 |
| 170 | 7/ 8/73 | 2/ 1/73 | 267 | 336 | 14 | M | 05 | 1 | WN206 |
| 171 | 24/ 8/73 | 24/ 4/73 | 264 | 326 | 7 | F | 07 | 1 | WN208 |
| 172 | 4/ 9/73 | 4/ 8/73 | 300 | 255 | 6 | F | 09 | 1 | WN209 |
| 173 | 6/ 9/73 | 15/ 7/73 | 290 | 361 | 12 | F | 04 | 1 | WN210 |
| 174 | 20/ 9/73 | 20/ 5/73 | 286 | 361 | 5 | M | 04 | 1 | WN211 |
| 175 | 21/ 9/73 | 10/ 9/73 | 290 | 356 | 7 | F | 04 | 1 | WN212 |
| 176 | 21/ 9/73 | 11/ 9/73 | 291 | 368 | 3 | F | 02 | 1 | WN213 |
| 177 | 3/10/73 | 3/ 7/73 | 275 | 378 | 4 | F | 01 | 1 | WN214 |
| 178 | 5/12/73 | 20/11/73 | 283 | 361 | 6 | F | 04 | 1 | WN216 |
| 179 | 29/ 1/74 | 22/ 1/74 | 270 | 325 | 5 | M | 07 | 1 | WN217 |
| 180 | 13/ 2/73 | / 8/73 | 270 | 357 | 7 | M | 03 | 1 | WN218 |
| 181 | 9/ 4/74 | 27/ 3/74 | 266 | 332 | 5 | F | 05 | 1 | WN220 |
| 182 | 11/ 9/74 | 28/ 8/74 | 267 | 367 | 6 | F | 03 | 1 | WN221 |
| 183 | 16/ 9/74 | 2/ 9/74 | 300 | 364 | 4 | M | 04 | 1 | WN222 |
| 184 | 5/10/74 | 5/ 9/74 | 268 | 390 | 8 | M | 01 | 1 | WN223 |
| 185 | 11/ 2/75 | 11/ 1/75 | 276 | 337 | 3 | M | 05 | 1 | WN225 |
| 186 | 21/ 2/75 | 27/ 1/75 | 273 | 345 | 10 | F | 04 | 1 | WN226 |

| Patient number | Date of diagnosis | Date of onset | Co-ordinates east | Co-ordinates north | Age | Sex | County | Diagnosis category | Reference number |
|---|---|---|---|---|---|---|---|---|---|
| 187 | 28/ 2/75 | 21/ 2/75 | 267 | 338 | 5 | M | 05 | 1 | WN227 |
| 188 | 17/ 3/75 | 17/ 1/75 | 293 | 396 | 16 | M | 02 | 1 | WN228 |
| 189 | 29/ 4/75 | 10/ 4/75 | 278 | 367 | 10 | F | 03 | 1 | WN229 |
| 190 | 21/ 5/75 | 1/ 5/75 | 266 | 332 | 6 | M | 05 | 1 | WN230 |
| 191 | 5/ 7/75 | 14/ 6/75 | 266 | 361 | 8 | M | 03 | 1 | WN234 |
| 192 | 5/ 7/75 | 21/ 6/75 | 267 | 344 | 5 | M | 05 | 1 | WN233 |
| 193 | 12/ 7/75 | 12/ 6/75 | 279 | 340 | 10 | M | 04 | 1 | WN235 |
| 194 | 4/ 8/75 | 20/ 4/75 | 298 | 268 | 13 | F | 09 | 1 | WN237 |
| 195 | 25/ 8/75 | 2/ 7/75 | 272 | 370 | 6 | M | 01 | 1 | WN240 |
| 196 | 28/ 8/75 | / 4/75 | 310 | 387 | 6 | M | 02 | 1 | WN241 |
| 197 | 9/ 9/75 | 7/ 9/75 | 265 | 335 | 12 | M | 05 | 1 | WN242 |
| 198 | 18/10/75 | 1/10/75 | 310 | 387 | 4 | M | 02 | 0 | WN244 |
| 199 | 13/11/75 | 2/10/75 | 279 | 339 | 5 | M | 04 | 1 | WN247 |
| 200 | 24/11/75 | 24/10/75 | 258 | 350 | 5 | F | 03 | 1 | WN246 |
| 201 | 12/12/75 | 12/ 8/75 | 327 | 383 | 6 | M | 10 | 1 | WN248 |
| 202 | 31/12/75 | 4/10/75 | 277 | 379 | 7 | F | 01 | 1 | WN250 |

*Codes*

*County:*

01 Koboko
02 Aringa
03 Maracha
04 Terego
05 Ayivu
06 Madi
07 Vurra
08 Okoro
09 Padyere
10 Jonam

*Diagnosis Category:*

0 Clinical diagnosis only
1 Histological proof